# Lead Responses and Tolerance Mechanisms of *Koelreuteria paniculata*: A Newly Potential Plant for Sustainable Phytoremediation of Pb-Contaminated Soil

**DOI:** 10.3390/ijerph192214968

**Published:** 2022-11-14

**Authors:** Rongkui Su, Tianzhi Xie, Haisong Yao, Yonghua Chen, Hanqing Wang, Xiangrong Dai, Yangyang Wang, Lei Shi, Yiting Luo

**Affiliations:** 1 School of Environmental Science and Engineering, Central South University of Forestry and Technology, Changsha 410004, China; 2 PowerChina Zhongnan Engineering Corporation Limited, Changsha 410004, China; 3 School of Civil Engineering, Central South Forestry University, Changsha 410018, China; 4 Hunan Engineering Research Center of Full Life-Cycle Energy-Efficient Buildings and Environmental Health, Changsha 410018, China; 5 College of Geography and Environmental Science, Henan University, Kaifeng 475004, China; 6 College of Environmental Engineering, Henan University of Engineering, Zhengzhou 451191, China; 7 Business College, Hunan First Normal University, Changsha 410205, China

**Keywords:** phytoremediation, Pb stress, *K. paniculata*, physiological response, tolerance mechanism

## Abstract

Phytoremediation could be an alternative strategy for lead (Pb) contamination. *K. paniculata* has been reported as a newly potential plant for sustainable phytoremediation of Pb-contaminated soil. Physiological indexes, enrichment accumulation characteristics, Pb subcellular distribution and microstructure of *K. paniculata* were carefully studied at different levels of Pb stress (0–1200 mg/L). The results showed that plant growth increased up to 123.8% and 112.7%, relative to the control group when Pb stress was 200 mg/L and 400 mg/L, respectively. However, the average height and biomass of *K. paniculata* decrease when the Pb stress continues to increase. In all treatment groups, the accumulation of Pb in plant organs showed a trend of root > stem > leaf, and Pb accumulation reached 81.31%~86.69% in the root. Chlorophyll content and chlorophyll a/b showed a rising trend and then fell with increasing Pb stress. Catalase (CAT) and peroxidase (POD) activity showed a positive trend followed by a negative decline, while superoxide dismutase (SOD) activity significantly increased with increasing levels of Pb exposure stress. Transmission electron microscopy (TEM) showed that Pb accumulates in the inactive metabolic regions (cell walls and vesicles) in roots and stems, which may be the main mechanism for plants to reduce Pb biotoxicity. Fourier transform infrared spectroscopy (FTIR) showed that Pb stress increased the content of intracellular -OH and -COOH functional groups. Through organic acids, polysaccharides, proteins and other compounds bound to Pb, the adaptation and tolerance of *K. paniculata* to Pb were enhanced. *K. paniculata* showed good phytoremediation potential and has broad application prospects for heavy metal-contaminated soil.

## 1. Introduction

In recent years, more attention has been paid to environmental pollution [1,2,3]. Heavy metal pollution is one of a number of environmental problems worldwide [4]. In 2014, total Pb production from mines around the world was 5.517 million tons. China owned the biggest share of 2.8533 million tons, accounting for 51.71%; which has been growing at a consistent rate of 2.5% per year [5]. At present, exposure to Pb slag poses an incalculable potential threat to nearby ecosystems [6,7]. The damage dealt by excessive accumulations of Pb, which brings about the reduction of biodiversity and the contamination of farmland and groundwater, should not be underestimated [8,9]. After heavy metal Pb enters the human body, it can cause certain damage to the nervous and reproductive systems of the body [10]. Long-term exposure to high levels of Pb may lead to atrophic gastritis, Alzheimer’s disease, and other symptoms; even death in severe cases [11,12]. Lanpear et al. [13] found that the number of deaths caused by high Pb blood content accounted for 18% of the total causes of death (IAA) using 1.43 × 10^4^ causes of death investigation reports; the number of people who could die from excessive Pb intake is 400,000 every year. Traditional physical and chemical remediation technologies (soil replacement technology, leaching technology, heat treatment technology, electric remediation technology, vitrification technology, chemical fixation technology, etc.) have disadvantages, such as high cost and secondary pollution [14,15,16,17,18,19,20,21]. Therefore, the remediation of contaminated soil in tailing areas by phytoecology has attracted increasing attention [22,23,24].

Phytoremediation techniques offer various merits, such as being eco-friendly, cost-effective, and having long-term benefits [25,26,27]. They could be ideal solutions for heavy metal-contaminated soil. Recently, the high enrichment properties of hyperaccumulators have been extensively studied in phytoremediation [28,29,30]. However, most plant species studied are annual herbaceous plants. Herbaceous hyper-enriched plants do have outstanding enrichment advantages, but they also have slow growth and limited biomass [31], which fails to provide an overall effective restoration of the herbaceous plants (e.g., *Thlaspi* sp.). Therefore, an urgent exploration of other plant species with advantages in terms of rapid growth and biomass growth is needed.

Phytostabilization mainly focuses on woody plants with heavy metal tolerance, and whose enrichment concentration is less than that of hyperaccumulators. However, their huge biomass and biological root systems formed by soil micro-organisms reduce the effective form of heavy metals, and they can reduce the migration and spread of heavy metals, especially in largely contaminated paddies [32]. As an inexpensive, highly efficient, and green technology, phytostabilization has been widely considered for its potential research and practical repair applications. Yet, additional research is still needed to determine a shortlist of plants that are resistant to heavy metals for the ecological restoration of contaminated areas [33].

*Black Locust* [34], *Platanus acerifolia* (Ait.) Willd. [35], and *Poplar* [36] have been proven to be effective in tailings remediation. As a widely distributed, fast-growing tree species in most provinces of southern China [37], *K. paniculata* is used for landscaping, energy, timber, and as industrial material. It provides nutrients to the soil and encourages the growth of other plants [38]. *K. paniculata* could play an active role in restoring bare ground and reducing the spread of heavy metal pollutants in contaminated areas. In an earlier experimental selection of Pb-Zn slag plants to investigate tolerance, it was found that *K. paniculata* could survive in Pb-Zn slag and showed good phytoremediation compared to 18 other tolerant plants [39]. However, the physiological tolerance effects and enrichment tolerance mechanisms of *K. paniculata* to Pb remain unclear.

This study intended to: (1) explore the changes in physiological and physicochemical characteristics of *K. paniculata* under Pb stress, including biomass, plant height, root structure, chlorophyll content, and antioxidant enzyme activity; (2) analyze the enrichment mechanism of tolerance in *K. paniculata* by the content of Pb in each tissue region and each subcellular content ratio; and (3) clarify the tolerance and adaptation mechanism of *K. paniculata* to heavy metals Pb through TEM and FTIR analysis. This investigation will provide a theoretical basis and technical support for the phytoremediation of heavy metals Pb.

## 2. Materials and Methods

### 2.1. Experimental Materials and Design

Experimental seedlings were obtained from the nursery of Central South University of Forestry and Technology (CSUFT). Seedlings were picked in good growth condition and maintained at a height of around 12 cm. Experimental cultivation sand (river sand) was purchased from Changsha Red-Star Flower Market and Hoagland nutrient was purchased from Beijing Xi-qing Agricultural Technology Company. The experiment was started in May 2020 in the nursery base of CSUFT, Changsha, Hunan Province, which is located in the subtropical monsoon climate zone with obvious continental climate characteristics and an average temperature of 25 °C during the experimental period. River sand (2 mm) was used as a substrate for the experiment, which was soaked with 2% hydrochloric acid and rinsed with deionized water. The plants were moved into the sand and incubated for 7 days until the growth state was stable. The Pb stress concentrations for each group were as follows: CK (0 mg/L), A (200 mg/L), B (400 mg/L), C (600 mg/L), D (800 mg/L), E (1000 mg/L), and F (1200 mg/L). CK was the control sample. Three replicates were set up for each gradient, with two seedlings containing 5 kg of sand in each pot. The Pb stress solution (Pb(NO_3_)_2_ solution) was added twice a week (100 mL each time), and the whole stress process lasted for 10 weeks. All plants were harvested after 70 days of Pb stress.

### 2.2. Experimental Method

#### 2.2.1. Plant Height, Biomass, and Root Length

The height of the plant was measured from the soil surface to the top of the plant, and root length was measured by a root analysis system (WinRHIZO PRO, Regent Instrument, Québec, QC, Canada). After harvesting, plant samples were heated to 105 °C for 30 min, dried to a constant weight at 75 °C, and then Pb concentrations were determined with a flame atomic absorption spectrophotometer (FAAS, AA-7002, Thermo Fisher Scientific, Waltham, MA, USA) [40].

#### 2.2.2. Pb Changes in Subcellular Fractions in Each Tissue of the Plant

Referring to the method outlined by [41], with some modifications, the harvested plants were washed with water, soaked in Na_2_EDTA solution to remove surface heavy metals, and were later rinsed with distilled water. The homogenate was stirred with a cooled extraction buffer [250 mM sucrose, 1.0 mM DTT (C_4_H_10_O_2_S_2_) and 50 mM Tris-HCl pH = 7.5]. The homogenate was centrifuged for 30 s (3000 rpm) and precipitated as the cell wall fraction (F1); the supernatant was centrifuged for a further 30 min (10,000 rpm) and precipitated as the organelle fraction (F2); the supernatant was the soluble fraction (F3). All operations were carried out at 3 °C.

#### 2.2.3. Determination of Chlorophyll and Antioxidant Enzyme in Plant Leaves

The determination of chlorophyll content in leaves of *K. paniculata* was obtained by spectrophotometry. The determination of malondialdehyde (MDA) was obtained by thiobarbituric acid method. The determination of soluble protein content was obtained by Coomassie brilliant blue g250 [42]. Superoxide dismutase (SOD) activity was determined by the *azotetrazolium* (NBT) method, guaiacol oxidation, and UV absorption, while peroxidase (POD) and catalase (CAT) activities were also determined.

#### 2.2.4. Microstructure and Functional Groups Analysis of the Plants

Roots and stems of fresh plants were washed with ultrapure water, cut into pellets (2 mm × 2 mm × 2 mm), and stored in glutaraldehyde solution (2.5%) at 4 °C to be measured. They were sent to Central South University for measurement by TEM (Tecnai Spirit, FEI, Hillsborough, OR, USA). Functional group composition was analyzed by Fourier transform infrared spectroscopy (FTIR). Dried tissue samples were crushed into powder with a pulverizer, passed through a 200 mm sieve, and analyzed by FTIR (Thermo Scientific Nicolet-iS10, Waltham, MA, USA) in a range of 400–4000 cm^−1^. Measurements were conducted by Shanghai Yeake Detection Equipment Co., Ltd. (Shanghai, China).

### 2.3. Statistics Analysis

Data were statistically analyzed using Microsoft Office Excel 2016. Least significant difference (LSD) tests were performed for multiple comparisons. If the difference was significant, it was marked as (*p* < 0.05). Then, a Duncan’s test with a 5% probability was performed to test for treatment differences. Finally, all data were expressed as mean ± standard deviation (SD) of three replicate experiments (*n* = 3).

Bioconcentration factor (BCF) and transfer factor (TF) of heavy metals are calculated as follows [9,26]:

BCF = Pb content in plant/Pb content in soil(1)

TF = Pb uptake of aboveground parts of plants/Pb total uptake of plants(2)

## 3. Results

### 3.1. Effects of Lead Stress on Plant Height and Biomass

In this experiment, *K. paniculata* survived to 100% under each concentration of Pb stress. Under the Pb concentration of 200 mg/L, it attained the highest height and biomass (Table 1), with its average height and total biomass increasing by 123.81% and 113.80%, respectively, compared to CK. When the Pb concentrations were further increased and were higher than that of 600 mg/L, physiological indicators such as plant height and biomass inversely decreased with the increase of Pb concentrations. For the 1200 mg/L treatment group, the leaf biomass and root biomass decreased by 59.1% and 31.9%, respectively, compared to CK. Furthermore, the average plant height after exposure to 1200 mg/L was similarly lower than that of the control.

### 3.2. Effects of Lead Stress on Root Morphology

Changes in root morphology are shown in Table 2. The promotion effect was pronounced in group A. Compared with CK, group A achieved 26.1%, 11.6%, and 11.2% increases in total root length, root surface area, and the number of fine roots, respectively. However, the total root length, surface area, and the number of fine roots were inhibited with the increase of Pb stress; the average root diameter proportionally increased with the increase of Pb stress. Compared with group F, the total root length increased by 755.4%, the total surface area rose by 309.9%, and the average root diameter decreased by 45.5% in group A.

### 3.3. Enrichment Capacity of K. paniculata for Pb

The accumulation of Pb in all sections of *K. paniculata* proportionally increased with Pb stress concentration (Table 3), and there was an overall trend of root > stem > leaf in the plant tissues. In the groups A, B, C, D, E, and F, the average value of Pb total accumulation amount in the *K. paniculata* was 3.12 mg, 4.29 mg, 5.58 mg, 7.32 mg, 10.14 mg, and 11.59 mg, respectively. These results showed that the total accumulation amount of Pb in the *K. paniculata* is positively related to the level of Pb stress. Pb accumulation amounts in roots, stems, and leaves of *K. paniculata* also showed a similar trend. The root storage of Pb was high, up to 85.0–93.3%. The bioconcentration factor (BCF) of *K. paniculata* was inversely proportional to the concentration of Pb stress and showed a significant difference (*p* < 0.05). This indicated that the enrichment capacity of *K. paniculata* fell with the rise of Pb concentration, while the highest BCF reached 0.65 at 400 mg/L. However, the trend of the transfer factor (TF) was stable between 0.07 and 0.13, and the highest reached 0.13 at 400 mg/L Pb stress.

### 3.4. Physiological Indexes of Pb Stress in K. paniculata Leaves

**Figure 1:**
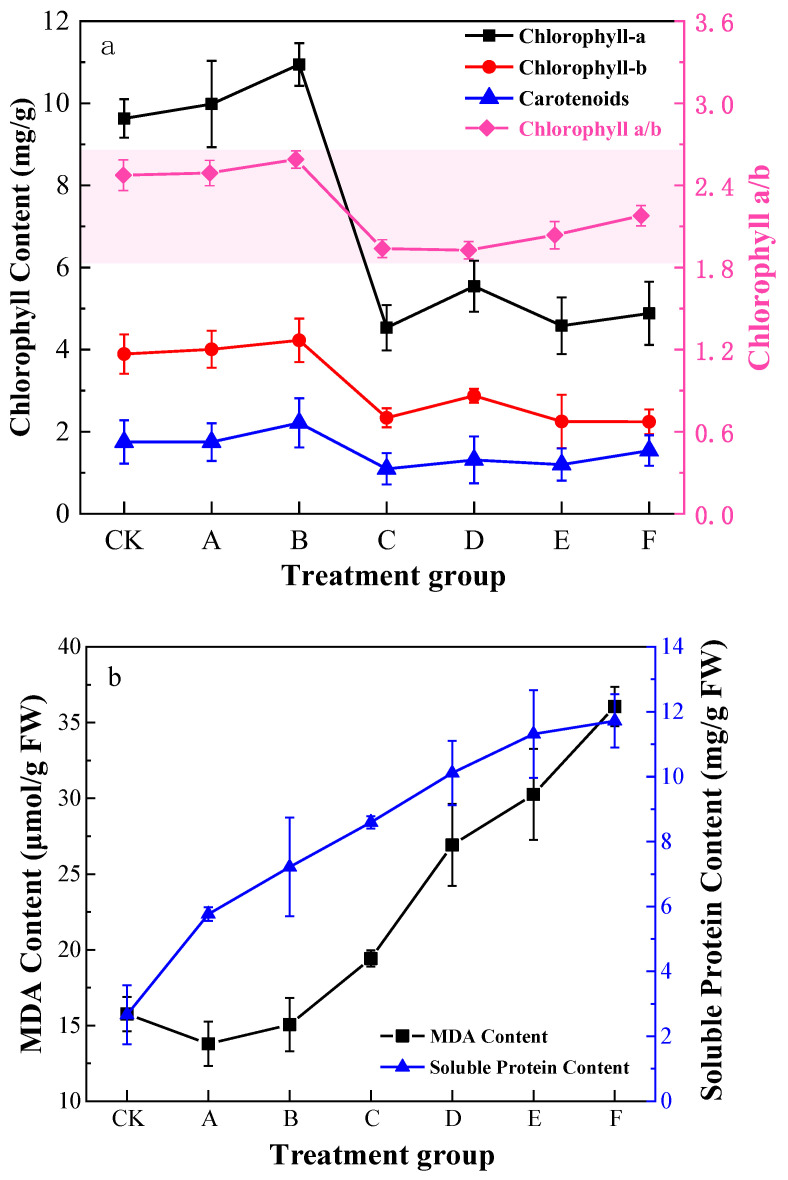
Pb stress on chlorophyll (**a**) and MDA content and soluble protein content (**b**) in the *K. paniculata* leaf under different treatment groups.

The chlorophyll content rose and then fell with increasing Pb stress concentration (Figure 1), while the chlorophyll content level reached peak at a stress concentration of 400 mg/L followed by a significant decrease; the changes of chlorophyll a/b in *K. paniculata* were consistent with the trend of total chlorophyll. The effect of chlorophyll content decreased with the increase of Pb concentrations.

Soluble protein content progressively rose with increasing Pb levels (Figure 1). The maximum amount in group E increased by about 423.0% compared to the minimum-level CK group. However, MDA content decreased and then increased. Compared with group CK, MDA slightly decreased in group A. Group F accumulated the highest amount of 36.06 µmol/g, which was 288.87% higher than the CK group. Membrane lipid peroxidation was impaired, but soluble proteins in *K. paniculata* leaves showed a coherent increase in response to MDA content, which counteracted some of the poor permeability caused by membrane lipid peroxidation.

**Figure 2:**
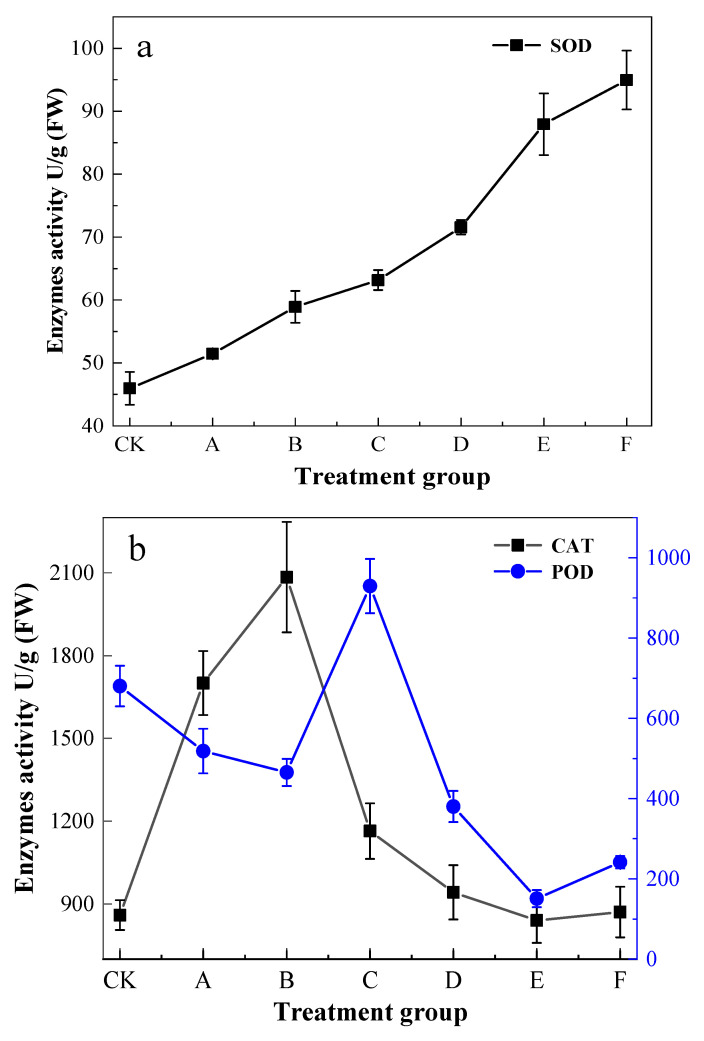
Effects of Pb stress on antioxidant enzymes activity in leaf blades. (**a**) SOD, (**b**) CAT and POD.

CAT activity showed a positive trend followed by a negative decline (Figure 2), with the highest point occurring in the B treatment group, reaching 2084.25 U/g (FW). Group E and group F were equivalent to the content of the CK group. The activity of SOD significantly increased (*p* < 0.05) with increasing levels of Pb exposure stress. The maximum of group F increased by about 206.6% compared to the CK group. The strongest POD activity appeared in group C, which showed a 142.9% increase in activity compared to CK, and the bottom point of group E was 74.5% lower than that of CK.

### 3.5. Effects of Lead Stress on Subcellular Distribution

**Figure 3:**
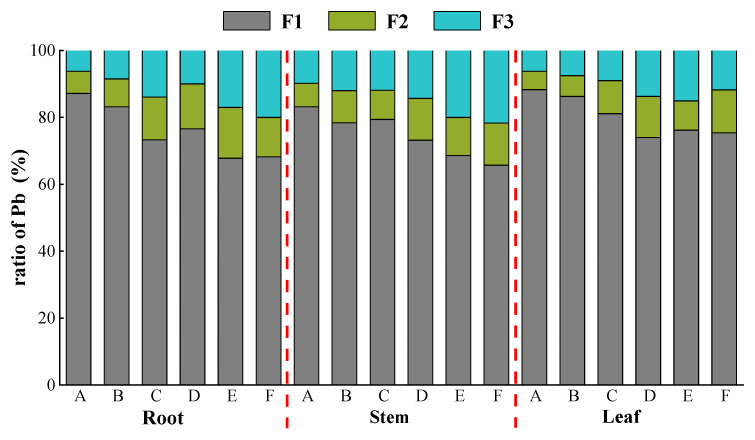
Distribution of Pb in different subcellular organs of *K. paniculata.* Note: F1 represents cell wall content fraction, F2 represents organelle content fraction, and F3 represents soluble component content fraction.

Under various concentrations of Pb stress, the overall trend showed F1 > F3 > F2 (Figure 3), with a large enrichment of Pb in F1 (68.2–87.2%). *K. paniculata* minimized the toxicity of Pb mostly via storage of Pb in the weakly active site cell wall fraction. As the concentrations of Pb stress increased, the proportion of Pb in the cell wall (F1) gradually declined, and the subcellular distribution shifted to the soluble fraction (F3) as a whole.

### 3.6. Effects of Lead Stress on K. paniculata Microstructure

**Figure 4:**
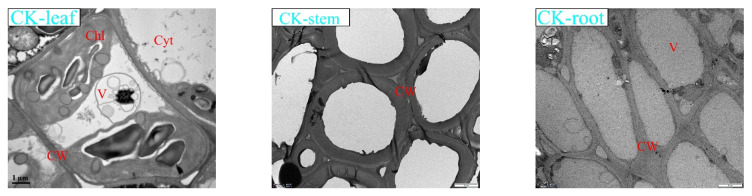

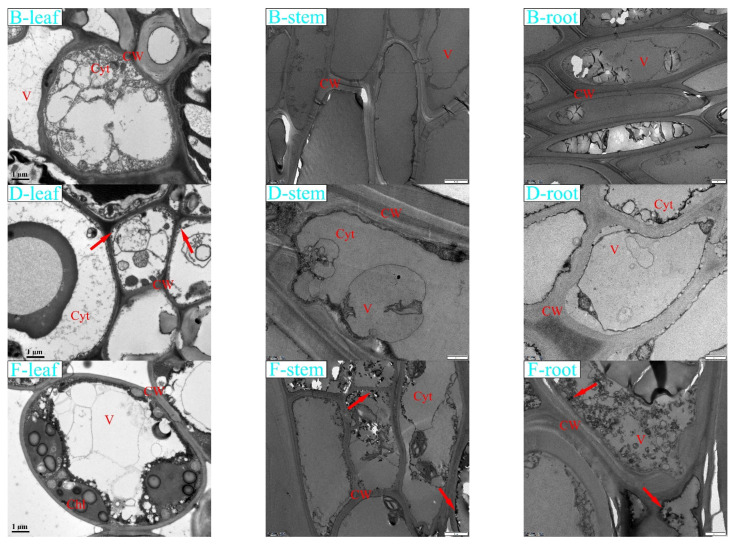
The effects of Pb stress on plant microstructure. Note: Pictures of root, stem, and leaf cells of *K. paniculata* seedlings in four treatments taken by TEM, CK treatment group, B treatment group (400 mg·L^−1^), D treatment group (800 mg·L^−1^), and F treatment group (1200 mg·L^−1^). CW: cell wall; SG: starch granules; Cyt: cytoplasm; V: vacuoles; Chl: chloroplast.

Transmission electron microscopy (TEM) was used to photograph the microstructure of each tissue of *K. paniculata* under the treatment of CK (0 mg/L), low concentration treatment group B (400 mg/L), medium concentration treatment group D (800 mg/L), and high concentration treatment group F (1200 mg/L) (Figure 4). The comparative analysis showed that in group CK of roots, stems, and leaves, the intercellular arrangement was regular, overall cell structure and cell membrane were intact, and no impurity materials filled the intercellular space. Roots, stems, and leaves were less affected by heavy metal hazards in group B. There was no obvious accumulation of impurities in the tissue cells. In the root, stems, and leaves of treatment group D, the suspected metal Pb accumulated inside the root cells and adhered to the cell walls, the membrane tissue was distorted and deformed, and the cell walls showed some damage but were able to maintain normal cell morphology. Pb granules may have precipitated in the cell wall and cytoplasm, as black granules were different from starch granules (SG) [43].

Conditions in leaf cells were slightly better than in stem cells, and no significant accumulation of Pb was found. In group F, water loss of the vesicles in *K. paniculata* root cells resulted in the separation of the protoplasm layer from the cell wall, while a large number of fine black particles accumulated in the cell wall and intercellular spaces inside the cells. The stem cells were locally and structurally damaged, and a small amount of fine black particles were found in the intercellular space and cell wall, which were significantly less than those in the roots. Pb accumulation in *K. paniculata* leaves was fair, with swollen chloroplasts and uneven lamellar structure, but cell morphology was somewhat affected.

### 3.7. FTIR Analysis of Functional Group Composition of K. paniculata Tissue

**Figure 5:**
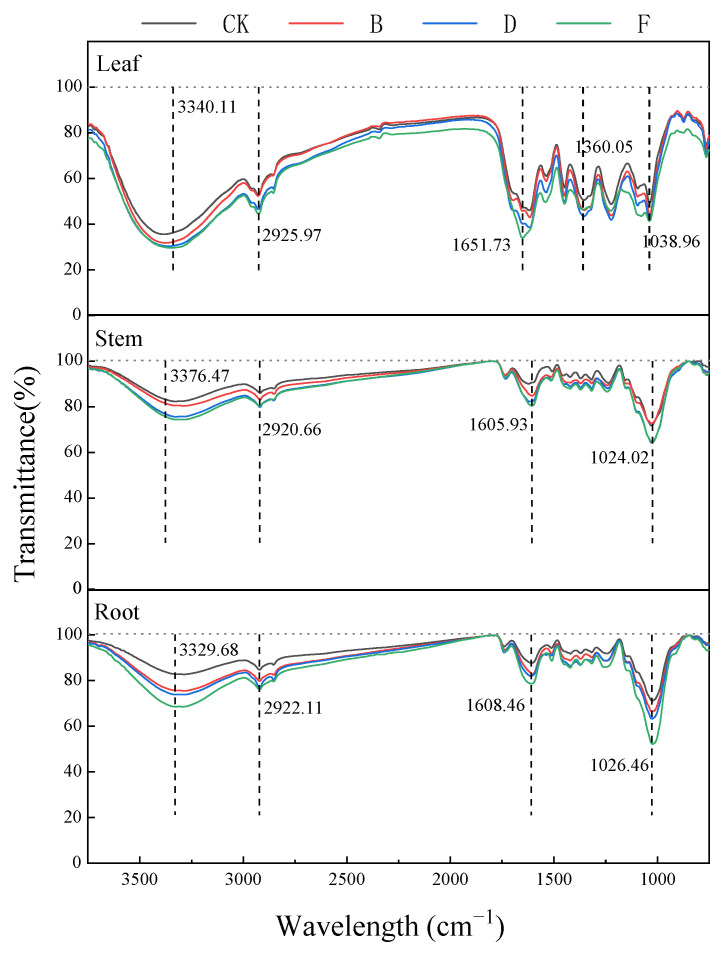
The functional groups of *K. paniculata* tissues on Pb stress at different concentrations. Note: CK treatment group, B treatment group (400 mg·L^−1^), D treatment group (800 mg·L^−1^), and F treatment group (1200 mg·L^−1^).

Compared with group CK, the FTIR spectral peak shape of *K. paniculata* tissue cells in other groups remained similar, but their transmittances showed significant differences at 3340 cm^−1^, 2920 cm^−1^, 1630 cm^−1^, and 1030 cm^−1^ (Figure 5).

At the vicinity of 3340 cm^−1^, there was a strong absorption with a rounded and blunt peak band associated with the superposition of the stretching vibration of O-H and the stretching vibration peaks of amino acids and protein amino groups (N-H); the change in absorbance at this point being mainly from changes in carbohydrates, such as cellulose, hemicellulose, and polysaccharides. The small and sharp absorption peak near 2920 cm^−1^, which represented -CH, was mainly from protein, cellulose, and pectin in the cell wall. The absorption peak at 1610 cm^−1^ was mainly from -COO and C=O stretching vibrations in amino acids, peptides, and proteins. The absorption peak near 1040 cm^−1^ was related to the stretching vibrations of S=O, mainly from polysaccharide carbohydrates [44]. The transmission of absorption peaks at 3420, 2920, 1630, and 1040 cm^−1^ decreased or shifted with increasing concentrations. These changes were evident in root cells, followed by leaves and stems, with relevant functional groups in the cell wall and soluble fraction, indicating that the cell wall and soluble fraction are important storage sites for Pb [45].

## 4. Discussion

### 4.1. Effects on the Growth of K. paniculata under Pb Stress

Growth morphology and biomass changes are the ultimate external form of plant growth adapted to the environment. Heavy metal stress inhibits plant growth, resulting in smaller leaf area, dwarfism, and reduced plant biomass [46]. In general, plants have a toxic response to a low level of heavy metal, and the biotoxicity of heavy metal is 10–100 times higher in hydroponic tests than in soil tests [47]. In this test, Pb concentrations below 400 mg/L accelerated the growth of *K. paniculata* compared to the CK. When Pb concentrations were above 600 mg/L, growth was significantly inhibited. However, plants did not show mortality, but adopted a set of physiological and biochemical tolerance changes to tackle the harm from Pb.

The roots serve as the site of direct exposure to heavy metals. In this experiment, the number of fine roots decreased with increasing stress concentration, the average root diameter decreased, and the specific surface area decreased in response to Pb stress. However, under growth-promoting stresses of 200 and 400 mg/L, the number of fine roots increased, and the specific surface area did not significantly change. It could be that *K. paniculata* actively adapts to Pb stress by increasing biomass and root share in adversity, improving nutrient and water extraction capacity. The increase in mean root diameter may provide protection of the root cells from heavy metal toxicity by increasing the thickness of the epidermis and non-protoplast barrier [48]. At the same time, root endothelium strengthening and the Kjeldahl band barrier are also essential mechanisms of root resistance to heavy metal ions [49]. Usage of physical and physiological avoidance strategies to intercept most of the heavy metal contaminants in the roots showed that *K. paniculata* can adapt to Pb contamination to some extent.

The root zone in *K. paniculata* accumulated more than 80% of the Pb. Studies have shown that plants have high metal tolerance, and can usually accumulate and fix large amounts of heavy metals in their roots, weakening the toxic effects on the plant body by preventing them from transferring from the subsurface to the ground [1]. Overall, it seems that the transport of all parts of *K. paniculata* is relatively small, no more than 10%, and its above-ground parts have a weaker ability to accumulate Pb than hyperaccumulator plants; however, with its huge biomass, the amount of enrichment should not be underestimated. Meanwhile, *K. paniculata* roots stressed by the high Pb concentration (1200 mg/L) accumulated 3200 mg/kg of Pb, and no mortality occurred, which is sufficient to verify the high tolerance property of *K. paniculata* to Pb.

### 4.2. Defense Mechanisms of K. paniculata Leaves under Pb Stress

High concentrations of Pb stress inhibited plant leaf growth, and this decreasing trend may be related to the production of reactive oxygen species (ROS) [50]. SOD, POD, and CAT are essential components of the plant antioxidant system, scavenging excess O_2_^-^ and H_2_O_2_ and their damage, inhibiting enzyme activity, poisoning membrane lipid peroxidation, and impairing the normal osmoregulatory capacity of cells [51]. In this study, enzyme activity did not show a single decline in *K. paniculata* leaves. However, the CAT and POD activity of the leaves showed a trend of increasing and then decreasing, and they respectively reached the turning points of stress amounts of 400 and 600 mg/L. The results might be attributed to the stimulation of antioxidant enzyme activity of *K. paniculata* within the threshold of Pb stress, but as the stress concentration increased, the enzyme activity system of *K. paniculata* was disrupted, and the enzyme activity decreased. Similar to previous reports [52], SOD activity maintained an upward trend to remove the harmful effects of reactive groups in the plant. In conclusion, *K. paniculata* reduces the toxicity of peroxides mainly by increasing leaf SOD activity and maintaining CAT and POD activity. MDA content of *K. paniculata* leaves was at a stable level under Pb 200–400 mg/L treatments, and significantly increased after the toxic effects of Pb stress became apparent. This might cause changes in the membrane structure of the leaf cells [53,54]. While Pb stress causes membrane lipid peroxidation damage in leaves, it also induces a response of antioxidant enzymes, antioxidants, and other resistance mechanisms in *K. paniculata*, leaving the total antioxidant capacity of *K. paniculata* leaves at a high level and enhancing the overall antagonistic capacity of *K. paniculata*. This phenomenon, similar to the elevated soluble protein content, balances the osmotic potential between the cytoplasm and the vesicles, and promotes the removal of reactive oxygen species in *K. paniculata*. It enables the normal proceeding of physiological activities such as cellular metabolism in *K. paniculata*, maintains the osmotic balance of cells, and protects them from the toxicity of heavy metals [55]. It may be one of the main resistance mechanisms in *K. paniculata*.

Chlorophyll content reflects the plants’ photosynthetic capacity and directly affects their growth and development [56]. Leaves in the treatment group above 1000 mg/L showed localized yellowing and black spots, similar to the response of many heavy metal-tolerant plants after suffering from excessive stress, such as *Robinia pseudoacacia* [57] and *Paulownia fortunei* [40], etc. The toxicity of Pb may affect the synthesis of prochlorophyll reductase and amino-γ-ketovaleric acid, hindering the chlorophyll synthesis process and leading to a decrease in chlorophyll content [58]. However, high chlorophyll a/b ratios suggest that the greater the stacking of cystoid bodies, the lower the photoinhibition and the more efficient plants are at using sunlight energy [59]. In this study, the a/b ratio content of stressed plants still fluctuated between 78.14% and 104.86% compared to the CK group. Despite causing some damage, *K. paniculata* was still able to maintain its growth by stabilizing chlorophyll a/b values to improve light energy use efficiency under high levels of Pb stress.

### 4.3. Microcosmic Structure Response of K. paniculata under Pb Stress

Cell wall fixation and vacuole-dominated distribution of soluble components are two main ways to detoxify heavy metals in plants [60,61]. The same conclusion was obtained in our experiment, and the distribution pattern of Pb in the subcellular fraction of *K. paniculata* was seen in the subcellular fraction of *K. paniculata* tissues, as well as in the relationship of cell wall > soluble fraction > organelle content fraction.

In all tissues of *K. paniculata*, the concentration of Pb distributed in the cell wall fraction always occupied the largest proportion, and the increase of Pb ions in the cell wall was even more obvious. The cell wall contains polysaccharides such as pectin, cellulose, hemicellulose, and protein, and many pro-metal ion coordination groups can be complex with positive-valent metal ions in an inactive state [60]. The cell wall plays an important role in the cumulative fixation of heavy metals and the reduction of their toxicity. After the concentration of Pb ions increases and the active sites in the cell wall are saturated and occupied, soluble fractions such as vesicles will absorb metal ions and thus reduce the toxic effect of Pb [62].

Employing transmission electron microscopy (TEM), the root cells of *K. paniculata* under Pb stress were observed: cell walls in the plants of group CK were smooth and well-organized; with the increasing concentration of Pb, the distribution of substances in each cell tissue became well-defined, and apparent aggregation effects were observed in the soluble components of the cell wall and vesicles. In addition, cell interstitial space also occupied a certain proportion of the content in the high concentration group F. The cell wall is the first barrier for extracellular substances to enter the cell. It contains a variety of polysaccharides and is rich in carboxyl, aldehyde, amino, and other metal-friendly coordination groups, which can be easily complexed with and immobilized by heavy metals [44,63]. When the heavy metal ions bound to the cell wall reach saturation, the thick wall of Pb ions interwoven by the cell wall dextran can block the most harmful heavy metal outside the cell and stay in the interstitial cell space. Depending on the loading level, excess heavy metal ions loaded on the cell wall leach into the cell and are transferred to the vesicles, where they are complex with organic acids and inorganic salts [64].

Fourier transform infrared spectroscopy (FTIR) is a technique for structural analysis based on the vibrations of functional groups and polar bonds in compounds [65,66]. In this experiment, 2920 cm^−1^ of -CH, -COO, and 1630 cm^−1^ of C=O may be more associated with the breakage of polysaccharide-rich peptide chains in the cell wall, combined with the widespread carboxylic acid and ketone groups in their secreted polysaccharides to reduce the toxicity of Pb to *K. paniculata* [67]. Compared to CK, especially for treatment B, the root promotion effect may be attributed to the increased secretion of amino acids and carboxylic acids in the cell wall and vesicles of *K. paniculata*, which increased the binding of polyester polysaccharides and heavy metals, and incidentally promoted the plant growth. This is consistent with the promotion of plant growth at low concentrations [68].

When stress levels increased, the peak absorbance of the above wavelength showed a downward trend. It could be that the hydroxyl group of the rhizome and the stem cell wall was complexed with Pb and the saturation of the hydrogen bond decreases. At the same time, high Pb stress inhibited the root secretion of *K. paniculata*, organic acids, amino acids, and polypeptide substances, and protein and root transport channels were affected. The absorbance of rhizome relative to leaf was significantly reduced in each peak spectrum change trend of 1030 cm^−1^ value in the infrared spectrum of leaf tissue with the increase of Pb treatment concentration, and finally tended to stabilize. As the Pb concentration increased, the level of cell membrane peroxidation was deepened, and the peroxide products of aliphatic ketones accumulated in the leaves to enhance the resistance of plates to Pb, which caused the increase in 1065 cm^−1^ values. The comparative analysis of the variation trend was 1030 cm^−1^. When roots, stems, and leaves are under high Pb stress, leaf tissue can still show specific Pb adaptability. It is speculated that some of the toxicity matter might be left in the root in exchange for reducing the toxicity in other parts to ensure the relative balance of the whole plant’s physiological metabolism, which is consistent with the physiological indexes of a plant root system.

## 5. Conclusions

Pb toxicity symptoms can affect physiological responses and inhibit plant growth and development. In this study, *K. paniculata* was found to be a highly Pb-tolerant woody plant. Under heavy metal Pb stress, the protective stress response of various antioxidant enzymes was obvious, and the threshold for stress damage production was high. Pb concentrations above 600 mg/L caused a decrease in various physiological indicators and inhibited plant growth, but the survival rate was not affected. Pb is mainly stored in the roots, and the local avoidance mechanism blocks the overall plant damage by optimizing the physiological and biochemical properties of the roots. In contrast, the fixation and sequestration of Pb by the cell wall and vesicles of root cells are the main pathways of Pb resistance in *K. paniculata*. As concentrations of Pb stress rose, the increase in soluble fractions acted as a fixation of Pb and the proportion of Pb content in the organelles. Microscopic TEM images showed slight damage to low and medium concentrations of Pb. However, at high concentrations of stress, a large amount of Pb was observed to accumulate in the cell wall, vesicles, and cell voids, breaking the structure of the cell wall and leading to the entry of Pb into the interior of the cell, which then disrupted the function of subcellular structures. At the same time, Pb stress increased the number of groups such as -COOH and -CH_3_ in *K. paniculata*, which can form stable compounds with Pb, and reduced the toxic effect of Pb on *K. paniculata*. Thus, this study provided a theoretical basis for the phytoremediation of Pb-contaminated soil by *K. paniculata.*

## Figures and Tables

**Table 1:** Effect of Pb stress on growth parameters of *K. paniculata* seedlings.

Treatment Group	Average Height (cm)	Total Biomass (g)	Root Biomass (g)	Stem Biomass (g)	Leaf Biomass (g)
CK	42.54 ± 0.67 ^c^	9.71 ± 0.71 ^b^	4.98 ± 0.33 ^a^	2.64 ± 0.12 ^c^	2.08 ± 0.1 ^a^
A	52.67 ± 0.85 ^a^	11.05 ± 1.045 ^a^	5.28 ± 0.21 ^a^	3.60 ± 0.1 ^a^	2.17 ± 0.06 ^a^
B	47.95 ± 1.95 ^b^	10.14 ± 0.45 ^ab^	5.15 ± 0.38 ^a^	3.33 ± 0.16 ^b^	1.65 ± 0.11 ^a^
C	38.40 ± 1.01 ^d^	8.50 ± 0.54 ^c^	4.49 ± 0.16 ^b^	2.66 ± 0.15 ^c^	1.34 ± 0.17 ^a^
D	37.60 ± 2.0 ^d^	7.04 ± 0.59 ^d^	3.93 ± 0.14 ^c^	2.03 ± 0.15 ^d^	1.08 ± 5.26 ^a^
E	37.20 ± 0.85 ^d^	6.60 ± 0.37 ^de^	4.07 ± 0.3 ^c^	1.61 ± 0.04 ^e^	0.92 ± 0.4 ^a^
F	31.80 ± 1.39 ^e^	5.70 ± 0.3 ^e^	3.39 ± 0.28 ^d^	1.45 ± 0.78 ^e^	0.85 ± 0.18 ^a^

Note: Different lowercase letters represent significant differences in growth data at different processing concentrations (*p* < 0.05).

**Table 2:** Changes of root structure in seedlings of *K. paniculata*.

Treatment Group	Total Root Length (cm)	Total Root Surface Area (cm^2^)	Average Root Diameter (mm)	Number of Fine Roots
CK	3079.01 ± 169.49 ^b^	576.04 ± 115.39 ^a^	1.32 ± 0.80 ^e^	1621 ± 117.20 ^ab^
A	3883.7 ± 429.61 ^a^	665.97 ± 98.48 ^a^	1.21 ± 1.10 ^e^	1803 ± 187.19 ^a^
B	2705.99 ± 122.29 ^b^	558.89 ± 46.63 ^a^	1.46 ± 1.50 ^de^	1413 ± 188.56 ^b^
C	1621.44 ± 179.88 ^c^	396.78 ± 20.36 ^b^	1.73 ± 0.29 ^cd^	995 ± 169.14 ^c^
D	1040.36 ± 247.13 ^d^	330.42 ± 54.04 ^bc^	2.02 ± 0.08 ^bc^	641 ± 107.53 ^d^
E	872.65 ± 123.18 ^de^	308.16 ± 51.49 ^bc^	2.25 ± 0.37 ^b^	582 ± 27.78 ^de^
F	514.46 ± 83.001 ^e^	215.84 ± 24.76 ^c^	2.67 ± 0.27 ^a^	365 ± 50.10 ^e^

Note: The data are the average of three groups of parallel experiments (*n* = 3), and different lowercase letters represent significant differences at processing concentrations (*p* < 0.05).

**Table 3:** The heavy metal content and total accumulation amount of *K. paniculata* under Pb stress.

Treatment Group	Pb Content (mg/kg)			BCF	TF	Total Accumulation Amount (Average Value, mg)
	Root	Stem	Leaf			
A	540.67 ± 13.05 ^e^	59.88 ± 5.38 ^e^	20.85 ± 4.49 ^f^	0.5 ± 0.014 ^b^	0.07 ± 0.003 ^c^	3.12
B	743.54 ± 55.48 ^e^	106.76 ± 1.75 ^d^	64.157 ± 5.49 ^e^	0.65 ± 0.013 ^a^	0.13 ± 0.01 ^a^	4.29
C	1143.25 ± 186.31 ^d^	119.24 ± 19.86 ^d^	93.21 ± 3.29 ^d^	0.46 ± 0.05 ^b^	0.09 ± 0.01 ^b^	5.58
D	1738.04 ± 192.28 ^c^	165.84 ± 7.81 ^c^	143.88 ± 17.16 ^c^	0.4 ± 0.02 ^c^	0.08 ± 0.005 ^bc^	7.32
E	2338.69 ± 151.27 ^b^	280.78 ± 16.7 ^b^	188.22 ± 24.12 ^b^	0.36 ± 0.05 ^c^	0.08 ± 0.012 ^bc^	10.14
F	3187.87 ± 251.77 ^a^	389.46 ± 21.7 ^a^	253.11 ± 7.81 ^a^	0.36 ± 0.12 ^c^	0.07 ± 0.003 ^c^	11.59

Note: Different letters in the same column indicate significant differences in Pb content in *K. paniculata* under different treatments (*p* < 0.05).

## Data Availability

The authors confirm that the data supporting the findings of this study are available within the article.
